# Eurasian and Sub-Saharan African mitochondrial DNA haplogroup influences pseudoexfoliation glaucoma development in Saudi patients

**Published:** 2011-02-19

**Authors:** Khaled K. Abu-Amero, Vicente M. Cabrera, José M. Larruga, Essam A. Osman, Ana M. González, Saleh A. Al-Obeidan

**Affiliations:** 1 Ophthalmic Genetics Laboratory, Department of Ophthalmology, College of Medicine, King Saud University, Riyadh, Saudi Arabia; 2 Área de Genética. Departamento de Parasitología, Ecología y Genética. Facultad de Biología, Universidad de La Laguna (ULL). Avenida Astrofísico Francisco Sánchez, s/n. 38206 La Laguna (Tenerife), Spain

## Abstract

**Purpose:**

To investigate whether different mitochondrial DNA (mtDNA) haplogroups have a role on the development of pseudoexfoliation glaucoma (PEG) in the Saudi Arab population.

**Methods:**

The mtDNA regulatory region and coding regions comprising mtDNA haplogroup diagnostic polymorphisms were sequenced in patients with PEG (n=94), healthy matched controls (free of PEG; n=112) and a healthy Saudi Arab population group (n=810).

**Results:**

The Eurasian haplogroup T and the Sub-Saharan African Haplogroup L2 confer susceptibility to PEG, whereas the Eurasian haplogroup N1 was associated with reduced risk to develop PEG in the Saudi Arab population.

**Conclusions:**

Mitochondrial haplogroups T and L2 may play a role in the development of PEG in the Saudi Arabian population.

## Introduction

Pseudoexfoliation (PEX) syndrome is a generalized disorder of the extracellular matrix and currently represents the most commonly identified specific cause of open-angle glaucoma [1]. The pathologic process is characterized by the chronic, stable accumulation of abnormal fibrillar aggregates in the anterior segment and various extraocular tissues [2]. PEX syndrome is frequently associated with pseudoexfoliation glaucoma (PEG), which often has a more aggressive clinical course and worse prognosis than the more common primary open angle glaucoma (POAG) [3]. Early studies were in support of a genetic basis for PEX/PEG. Furthermore, pedigree analysis suggested maternal transmission, raising the possibility of mitochondrial inheritance or X-linked inheritance [4]. However, studies focused on functional candidate genes potentially involved in PEX [5,6] found only one significant association between a clusterin gene variant (*CLU* SNP rs2279590) and PEX in German patients that could not be confirmed in Italian patients. A latter genome-wide association study involving the same cohorts identified variants at contactin associated protein-like 2 gene (*CNTNAP2*) associated with PEX but again only in German patients [6]. It seems that, until now, the only consistent association between gene polymorphisms and PEX/PEG is that with relatively common sequence variants in the Lysyl oxidase-like 1 (*LOXL1*) autosomal gene. This association was first detected in Nordic European samples [7] and, since then, confirmed in samples with broad ethnic diversity [6,8-10]. However, the few gene polymorphisms found associated with PEX/PEG cannot explain the population incidence of these syndromes. It seems that the hereditary susceptibility detected could be better explained as the result of multigenic combinations in which mtDNA polymorphisms might also participate. In fact, in a recent study, it was found that mitochondrial haplogroup U was associated with a reduced risk to develop exfoliation glaucoma in the German population [11].

In Saudi Arabia about 10% of glaucoma patients are PEG [10] and in the eastern region of the Arabian Peninsula PEX accounts for 77% of all cases of open-angle glaucoma [12].

Similar to almost all non-African populations tested thus far, the “G” allele of both rs1048661 and rs3825942 SNPs were associated with the risk of PEG in the Saudi Arab population [10]. There are also studies of the effect of mitochondrial gene mutations and polymorphisms on PEG in Saudi Arabia. However, there was little evidence that potentially pathological mtDNA mutations played a significant role in PEG in Saudi Arabia [13]. In another study, assessing the role of mitochondrial haplogroups in glaucoma, also in the Saudi Arabian population, it was found that patients with haplotypes belonging to the preHV-1 (now named R0a) haplogroup were at higher risk of developing primary angle-closure glaucoma (PACG), but it could not detect any significant difference in haplogroup frequencies between PEG patients and controls [14]. However, the small sample size for PEG (n=29) in that study [14], and its subdivision into different mtDNA haplogroups, was clearly insufficient to perform robust statistical analysis. The aim of the present study was to assess the role of mtDNA haplogroups in PEG among Saudi patients. This aim will be achieved by comparing the mtDNA haplogroup frequencies in a PEG-patient cohort (n=94) with those of matched healthy controls (n=112) and with a large healthy sample (n=810) taken for population genetics purposes.

## Methods

### Study population

This study adheres to the tenets of the Declaration of Helsinki and all participants signed an informed consent. The study was approved by the College of Medicine, King  Saud University, Riyadh, Saudi Arabia ethical committee (proposal number 08–657). All study subjects were self identified as Saudi Arabian ethnicity. Subjects with clinically diagnosed PEG (n=94) and age and sex healthy matched controls, HMC, (n=112) were recruited into the study at King Abdulaziz University Hospital in Riyadh, Saudi Arabia.

All participants underwent a standardized detailed ophthalmic examination, which included visual acuity, measurement of intraocular pressure (IOP) by Goldmann applanation tonometry, slit lamp biomicroscopy, gonioscopy, and detailed examination of the lens and fundus. Patients with PEG were defined as those with clinical evidence of exfoliation material on the pupil margin or anterior lens surface and the presence of glaucomatous optic neuropathy with associated visual field defect in one or both eyes and documented IOP >22 mmHg in either eye. Saudi Arab subjects (n=112) with normal anterior segment and optic nerve examination, IOP <18 mmHg, and no clinical signs of exfoliation were recruited as control subjects.

A third large sample (n=810) of healthy Saudi Arabs (HSA), representing all five major Saudi Arabian provinces and were recruited previously for population genetics studies, was used for statistical comparisons. All individuals of this group were Saudi Arabs who reported no symptomatic metabolic, genetic, ocular disorders or any ophthalmic problem on an extensive questionnaire regarding family history, past medical problems, and current health. For those controls, their mother’s ancestral origin was established as a Saudi. The mtDNA haplogroup assignation for 552 subjects of this sample has been already published [15].

### DNA extraction

Five ml of peripheral blood were collected in EDTA tubes from all participating individuals. DNA was extracted using the illustra blood genomicPrep Mini Spin Kit from GE Healthcare (Buckinhamshire, UK), and stored at –20 °C in aliquots until required.

### Mitochondrial haplogroup assortment

All samples were amplified and sequenced for the mtDNA regulatory region hypervariable segments (HVSs) I and II using primers pairs L15996/H16401 and L16340/H408, respectively, as previously described [16]. Haplotypes were tentatively assorted into haplogroups according to their HVS diagnostic positions. This assortment was further confirmed, when necessary, by the analysis of coding-region diagnostic haplogroup polymorphisms (Table 1), following the most recent mtDNA haplogroup nomenclature [17]. To detect these polymorphisms, a fragment spanning the diagnostic position was amplified and sequenced using any of the 32 overlapping pairs of primers that cover the whole mtDNA genome, and the PCR and sequencing conditions previously published for each of them [16]. To keep adequate haplogroup sub-sample sizes within cohorts, mtDNA haplogroups were collapsed into larger haplogroup identities following a phylogenetic criterion [17].

**Table 1:** Haplogroup distribution in PEG, HMC, and HSA groups.

**Mitochondrial haplogroup**	**mtDNA coding position**	**PEG (n=94)**	**HMC (n=112)**	**HSA (n=810)**	**PEG vs HMC p value**	**PEG vs HSA p value**	**cPEG vs HMC p value**	**cPEG vs HSA p value**
H	C7028C	6 (6.4%)	4 (3.6%)	66 (8.1%)	0.350	0.550	0.442	0.344
R0a	T3847C	15 (16%)	11 (9.8%)	141 (17.4%)	0.187	0.725	0.053	0.726
J	T4216C	24 (25.5%)	28 (25%)	171 (21.1%)	0.930	0.324	0.646	0.103
T	T4216C; G15928A	11 (11.7%)	4 (3.6%)	53 (6.5%)	0.025	0.065	0.039	0.105
K	A3480G	4 (4.3%)	3 (2.7%)	27 (3.3%)	0.534	0.642	0.380	0.387
U	A12308G; G12372A	8 (8.5%)	19 (17%)	91 (11.2%)	0.073	0.423	0.103	0.626
Other R	C12705C	3 (3.2%)	4 (3.6%)	25 (3.1%)	0.881	0.956	0.610	0.695
N1	T10238C; G12501A	0	8 (7.1%)	66 (8.1%)	0.008	0.004	0.003	0.001
W	G15884C	0	0	6 (0.7%)	1.000	0.402	1.000	0.338
X	T14470C	2 (2.1%)	3 (2.7%)	20 (2.5%)	0.798	0.839	0.907	0.984
M1	C10400T; C12403T	3 (3.2%)	8 (7.1%)	25 (3.1%)	0.209	0.956	0.176	0.921
M(x M1)	C6371T	3 (3.2%)	5 (4.5%)	24 (3%)	0.638	0.902	0.392	0.747
L2	T10115C	10 (10.6%)	4 (3.6%)	35 (4.3%)	0.229	0.458	0.080	0.175
L(x L2)	C12705T; C10400C	5 (5.3%)	11 (9.8%)	60 (7.4%)	0.045	0.008	0.140	0.066

PEG: Pseudoexfoliation glaucoma patients; cPEG: Pseudoexfoliation glaucoma patients from this study (94) and those (29) from [14]; HMC: healthy controls (free of PEG, see methods); HSA: healthy Saudi Arabs (see methods for details). χ^2^ or Fisher exact tests were applied to investigate the association between having a certain haplogroup and the occurrence of PEG.

### Data analysis

Global comparison of haplogroup frequencies among patient and control cohorts were performed by analysis of molecular variance (AMOVA) among groups. Differences between groups were tested by pair-wise exact tests of sample differentiation [18,19], based also on haplogroup frequencies. In both cases the Arlequin 3.11 package was used. χ^2^ or Fisher exact tests were applied to investigate the association between having a certain haplogroup and the occurrence of PEG compared to controls.

## Results

Table 1 presents haplogroup frequencies in the PEG and HMC cohorts and in the general Saudi Arab population (HSA) sample. Table 1 showed that haplogroups R0a and J were particularly abundant in the three groups tested and this is previously known characteristic of the Arab population mtDNA Haplogrouping [15,20]. The prevalence of these two haplogroups combined was 41%, 35%, and 39% for PEG, HMC and HSA groups, respectively. In comparison to the HSA group, the lack of the Eurasian haplogroup N1 representatives, the low frequency of the Eurasian haplogroup U and the high frequency of the Eurasian haplogroup T and the sub-Saharan African haplogroup L2 were distinctive of the PEG cohort. The high frequency of haplogroup U was the most outstanding characteristic of the HMC cohort (Table 1). Congruently, global AMOVA analysis showed a significant haplogroup frequency heterogeneity among groups (p=0.023). Pair-wise exact tests between cohorts showed that this statistical heterogeneity is mainly due to particular differences between PEG versus HMC (p=0.016) and PEG versus HSA group (p=0.027) as haplogroup differences between both control groups did not reach statistical significance (p=0.178). χ^2^ or two tailed Fisher exact tests analysis of haplogroup frequencies in PEG patients versus HMC group indicate that haplogroups T and L2 were in significant excess in PEG patients compared to HMC (p=0.025 and p=0.045, respectively). When we performed the same analysis for the PEG patients versus HSA group, we noticed that haplogroups T and L2 were more abundant in the PEG patients compared to the HSA groups. Statistically, this abundance was significant for the L2 haplogroup (p=0.008) and near significance for the T haplogroup (p=0.065). Furthermore, the lack of Eurasian haplogroup N1 representatives in PEG patients, significantly contrasts with its moderate presence in both HMC (p=0.008) and HSA (p=0.004) control groups. However, the respective higher and lower frequencies found in HMC and PEG cohorts for the Eurasian haplogroup U was near significance, but did not reach statistical significance (p=0.073). These data could be interpreted as that haplogroups T and L2 associated with the risk of developing PEG in the Saudi population, while mitochondrial haplogroup N1 could be a protective or associated with reduced risk of developing PEG.

## Discussion

Despite early genetic studies indicating that maternal transmission could play an important role in PEX susceptibility [4], few studies have been performed to test the effect of mitochondrial background on PEX/PEG syndromes. In the present study, we found that carriers of the Eurasian mtDNA haplogroups T and the sub-Saharan African haplogroup L2 were at higher risk of developing PEG. In contrast, carriers of the Eurasian haplogroup N1 were at reduced risk to develop PEG.

Previously, we investigated the frequencies of mtDNA haplogroups in a 29 diagnosed PEG Saudi subjects [14]. Here we attempted to test whether the associations found here still hold when a composite PEG (cPEG) cohort was compared with both present work control groups (HMC and HSA). Since haplogroup N1 was also absent in the previously published PEG cohort, statistical significance rises when the cPEG cohort was tested against HMC (p=0.003) and against HSA (p=0.001). However in the test for haplogroup T involving cPEG versus HMC, although still significant (p=0.039), a lower p-value than the one found in the present study (p=0.025) was obtained. Again, testing cPEG vs HSA lacked statistical significance (p=0.105). Similar results were obtained for comparisons involving haplogroup L2, while the test cPEG versus HSA was near significance (p=0.066) significance disappears in the test involving HMC (p=0.140).

Recently, mtDNA haplogroup U was significantly associated with a reduced risk of developing exfoliation glaucoma in the German population, whereas haplogroup T was slightly over-represented in the glaucoma cohort when compared to controls. However, these differences did not reach statistical significance [11]. It is inquisitive that the trends for haplogroups T and U are the same in the Saudi Arab and the German populations with, respectively, higher and lower frequencies in PEG patients compared to their respective matched controls. However, whereas only haplogroup T based comparison reached significance in the Saudi Arabian population, haplogroup U comparison reach significance among Germans. N1 and L2 haplogroups could not be tested in the German population because, N1 haplogroup is very scarce in Europe and L2 is mainly confined to the African continent.

Most probably the Eurasian haplogroups T and U have, respectively, mild risk and protective effects in the development of PEG. Additionally, carriers of the Eurasian haplogroup N1, at least among the Saudi Arabian population, seem to be at significant reduced risk to PEG whereas those with the sub-Saharan African haplogroup L2 are associated with PEG susceptibility. Although all the coding diagnostic variants of haplogroups L2 and U are synonymous, there is a nonsynonymous transition, A13780T, in the root of haplogroup N1 that changes an Ile amino-acid to Val in the mitochondrial NADH dehydrogenase subunit 5. There is also a nonsynonymous transition, A4917G at the root of haplogroup T that changes an Asn amino-acid to Asp in the mitochondrial NADH dehydrogenase subunit 2. However, the fact that these variants are widely distributed in the healthy population, weaken the hypothesis of a direct involvement of these mitochondrial variants in the development of PEG. Nevertheless, we could not ignore a possible interaction with the nuclear-genome which may influence the development of PEG. In addition to geographic mtDNA haplogroup frequency differences, population structure might mask or enhance these types of associations. In fact, the mtDNA haplogroup associations with PEG found in this study were stronger when the PEG cohort was compared to the matched healthy controls (free of PEG by examination) than when it was compared to the Saudi Arabian global sample (HSA group).

In conclusion, we have found that T and L2 haplogroups confer susceptibility to PEG whereas N1 haplogroup is associated with a reduced risk to develop this illness. To know whether these associations are consistent in other geographic areas, sample sizes should be adequate to the relative frequencies of the mtDNA haplogroups involved in them. Additionally, the incorporation in the genome-wide association studies of the mtDNA SNPs that define the most important haplogroups worldwide would help to detect those nuclear-mitochondrial gene interactions that predispose or protect to illnesses with polygenic diseases such as PEG.
